# *Anenteotermescherubimi* sp. n., a tiny dehiscent termite from Central Africa (Termitidae: Apicotermitinae)

**DOI:** 10.3897/zookeys.793.28342

**Published:** 2018-10-29

**Authors:** Rudolf H. Scheffrahn, Yves Roisin

**Affiliations:** 1 Fort Lauderdale Research and Education Center, Institute for Food and Agricultural Sciences, 3205 College Avenue, Davie, Florida 33314, USA Institute for Food and Agricultural Sciences Davie United States of America; 2 Evolutionary Biology and Ecology, CP 160/12, Université Libre de Bruxelles, Avenue F.D. Roosevelt 50, 1050 Brussels, Belgium Université Libre de Bruxelles Brussels Belgium

**Keywords:** abdominal autothysis, enteric valve armature, soldierless new species

## Abstract

*Anenteotermescherubimi* Scheffrahn, **sp. n.** is described from workers and male imagos collected in Cameroon and Republic of the Congo. This is the smallest soldierless termite known from Africa. As with many soldierless and soil-feeding termite species, the enteric valve morphology is a robust and essential diagnostic character for *An.cherubimi*. Preserved workers display pre-autothysis morphology and the effects of abdominal autothysis.

## Introduction

In his revision of the soldierless termites of Africa, Sands (1972) described 60 species (51 new) among 16 new genera, all beginning with the letter “A”. All were previously placed in the genus *Anoplotermes* Müller. Sands (1972) described ten new species from his second-largest new genus, *Anenteotermes*. In his broader treatise on soil-inhabiting termite workers of Africa, Sands (1998) recapitulated his descriptions of *Anenteotermes* with one exception; he depicted the enteric valve armature (EVA) of “*Anenteotermes* new species” (plate 8, fig. 9). Sands (1998: 169) also noted “At least one new species awaits description from rain forest”. We herein describe this remarkable new *Anenteotermes* as *A.cherubimi* sp. n., the smallest known soldierless termite in Africa.

## Materials and methods

Preserved workers, stored in 85% ethanol, were positioned in a transparent petri dish filled with Purell® hand sanitizer (70% EtOH). Body sections and dissected guts were photographed as multi-layer montages using a Leica M205C stereomicroscope with a Leica DFC 425 module run with Leica Application Suite software version 3. Mandibles and EVA were mounted on slides with PVA mounting medium (Bioquip Products, Inc.) and photographed with a Leica CTR 5500 compound microscope using bright field lighting and the same montage software. Imagos were photographed in alcohol on sand. Terminology of the worker gut follows that of Sands (1972) and Noirot (2001). Measurements were obtained using an Olympus SZH stereomicroscope fitted with an ocular micrometer. All specimens described here are deposited in the authors’ collections under the accession numbers AFR1508 and AFR1282 for RHS and CGO060 for YR.

## Systematics

### Anenteotermes cherubimi

Taxon classificationAnimaliaBlattodeaTermitidae

Scheffrahn sp. n.

http://zoobank.org/12A5A940-6CDB-4B64-A710-C3B3B82CD832

**Figure 1.**
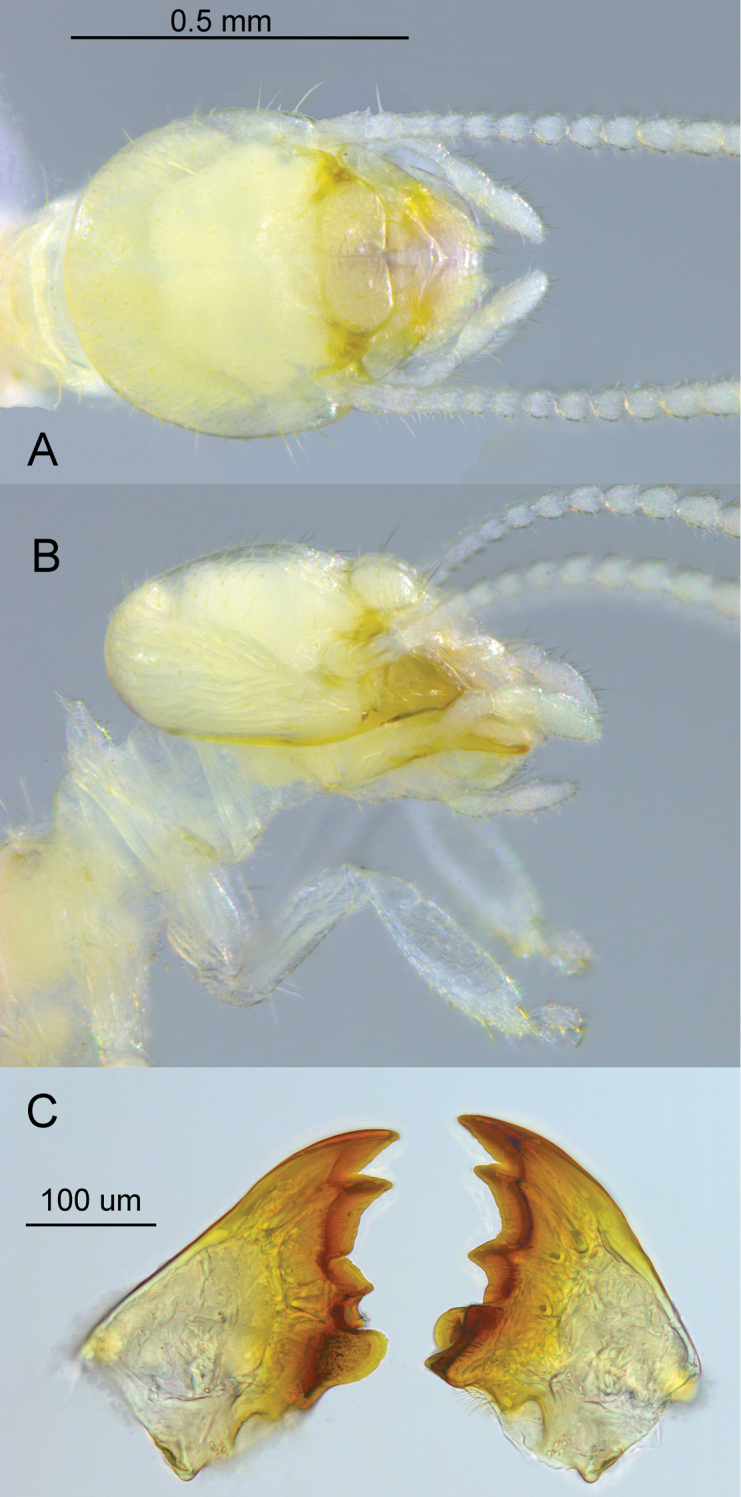
Worker of *Anenteotermescherubimi* sp. n. **A** dorsal **B** lateral view of head and prothorax **C** mandibles.

**Figure 2.**
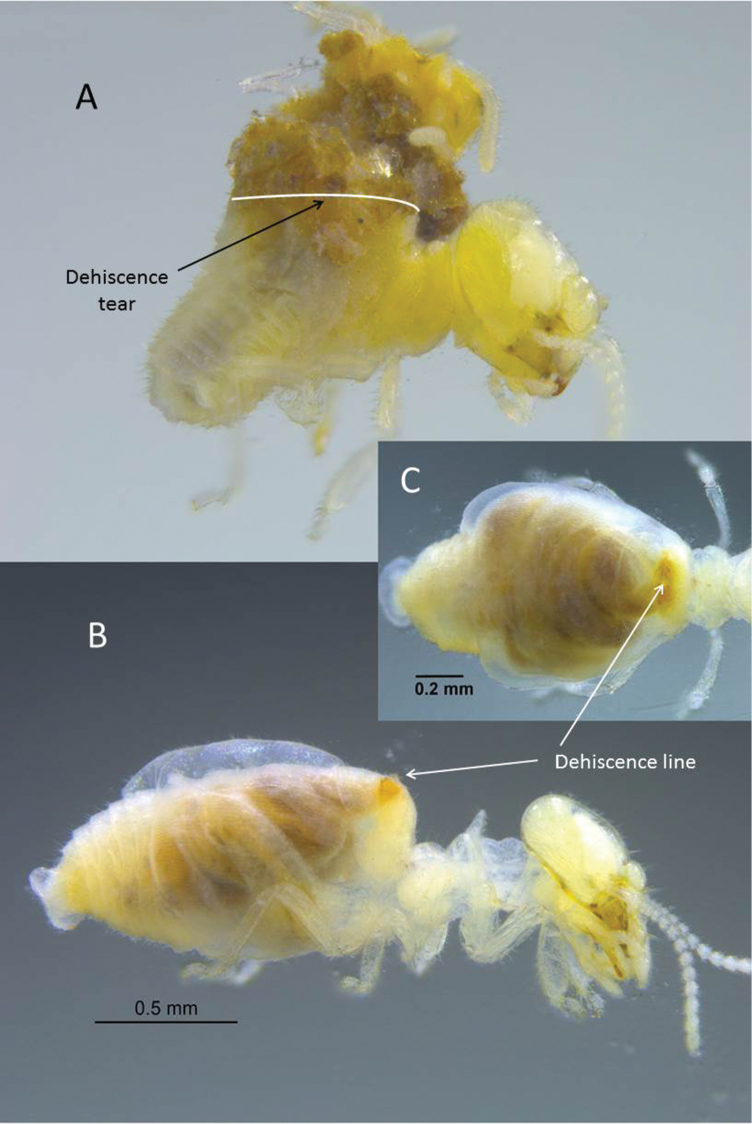
Worker of *Anenteotermescherubimi* sp. n. **A** Gut contents expelled after tear of dehiscence line. Dehiscence line slightly open in lateral **B** and dorsal **C** views.

**Figure 3.**
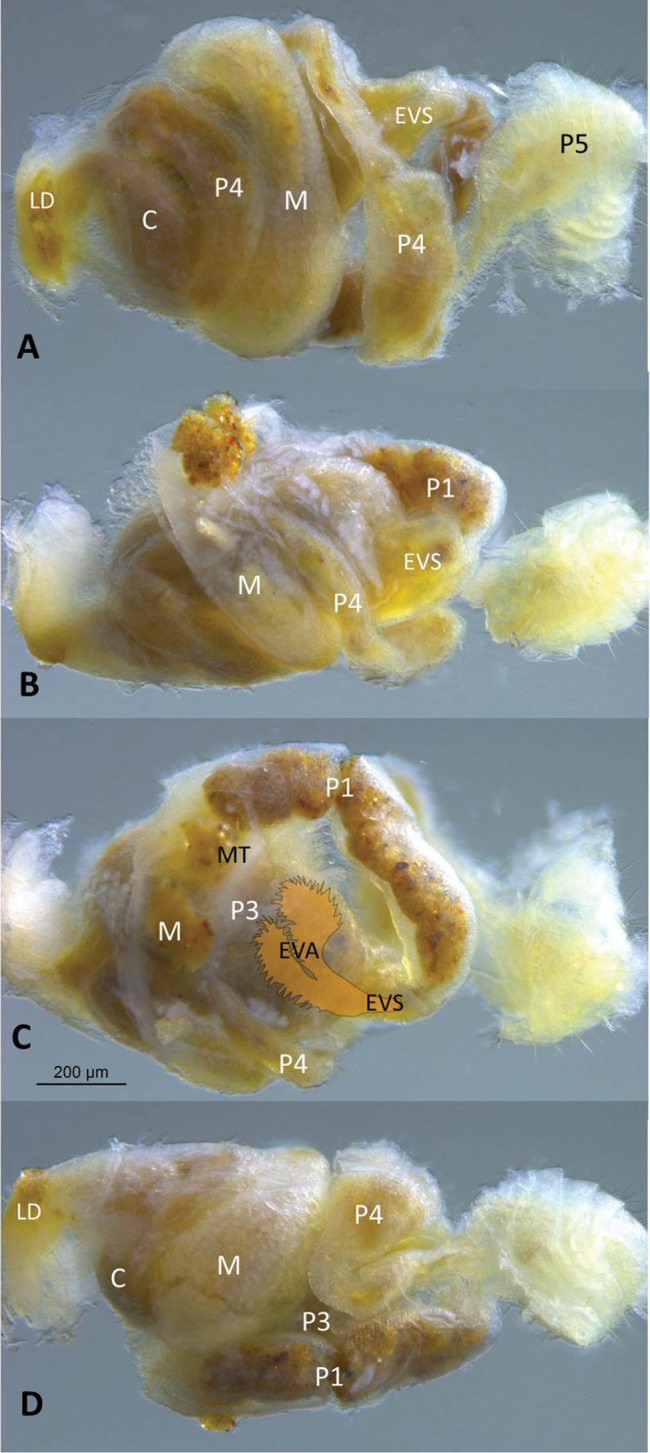
Gut of *Anenteotermescherubimi* sp. n. worker. **A** dorsal **B** right with area near mixed segment and mesenteric tongue (MT) torn (see Figure **1D**) **C** ventral with position of enteric valve armature (EVA) and enteric valve seating (EVS) drawing superimposed to scale **D** left views. Abbreviations: C = crop, LD = dehiscence line; M = mesenteron; P1, P3, P4, and P5 = proctodeal segments.

**Figure 4.**
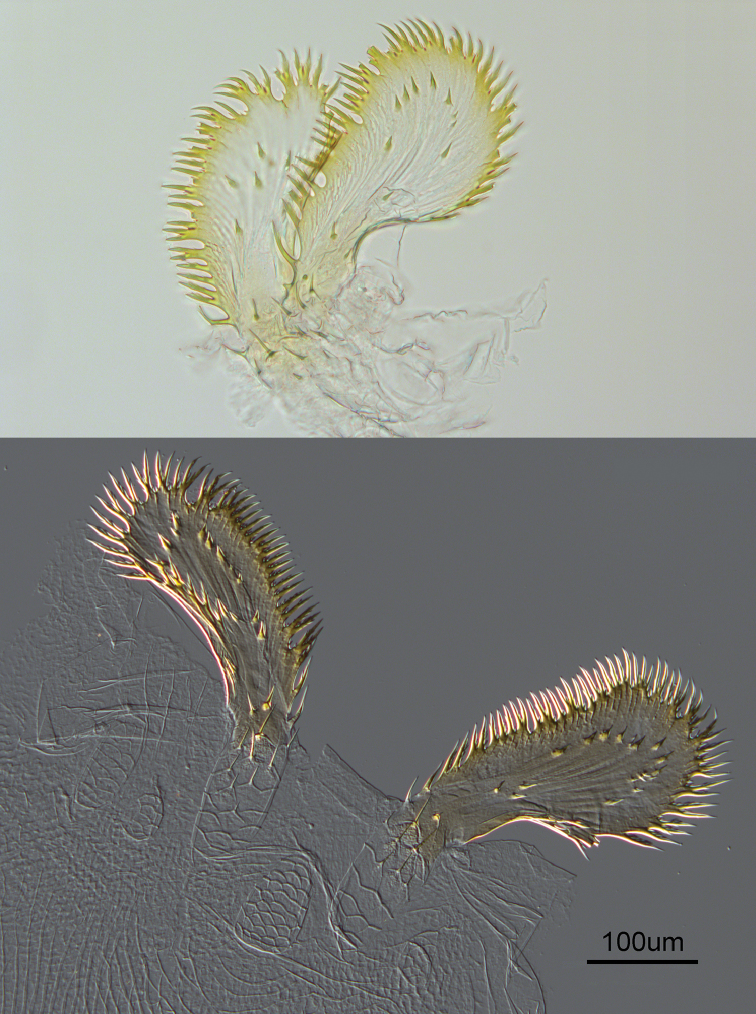
Enteric valve armature of *Anenteotermescherubimi* sp. n. worker. Top: paddles folded (see Figure **3C**); bottom: paddles fully extended.

**Figure 5.**
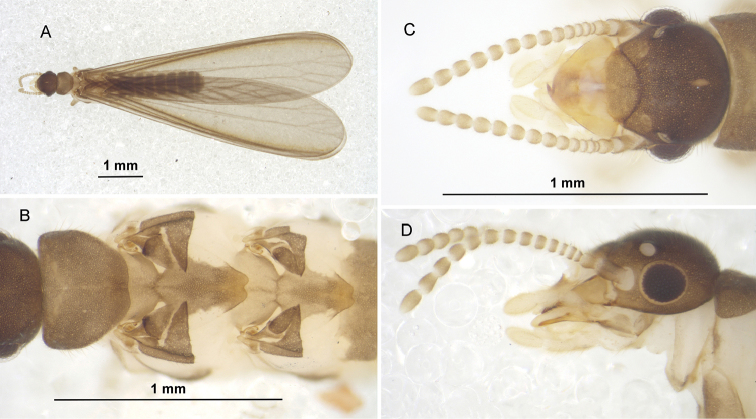
Male imago of *Anenteotermescherubimi* sp. n. **A** dorsal view **B** dorsal nota **C** dorsal **D** lateral view of head.

Figures 1 , 2 , 3 , 4 , 5

#### Material.

**Holotype.** Worker from colony UF no. AFR1508. University of Florida Termite Collection, Fort Lauderdale Research and Education Center, Davie, Florida. **Paratypes**. CAMEROON: Ebogo, slope above Nyong River (3.386, 11.466), 667 m elev., 23NOV2011, col. J. Křeček, AFR1282, two workers in capped plastic vial and approx. 25 workers desiccated in broken glass vial collected with *Orthotermes* sp. CAMEROON: Ebogo II (3.386, 11.682), 660 m elev., 10DEC2011, col. J. Šobotník, AFR1810, 4 workers, larvae. CONGO (Republic of): Mokabi SA (Groupe Rougier) logging concession (3.14658, 16.96377), 527 m elev., 8DEC2017, col. Y. Roisin, Accession no. CGO060, 7 male imagos and 11 workers in soil at base of tree in rainforest.

#### Type locality.

CAMEROON: Korup National Park (5.0045, 8.8635), 109 m elev., 5DEC2011, col. J. Křeček, UF no. AFR1508, 53 workers collected under stone.

#### Description of worker

(Figs 1–4, Table 1). Monomorphic, very small, approx. 2 mm. Head capsule yellowish, covered with approximately 100 setae of varying length (Figure 1A, B). Postclypeus moderately inflated, fontanelle indiscernible. Anterior margin of abdomen, in lateral view, raised vertically above metanotum and marked at apex by dehiscence line (Figure 2 A-C). Antennae with 13 articles; 23=4=56. Left mandible with apical and first marginal teeth well separated, long, and projecting well beyond line formed by third marginal tooth and molar prominence. A subsidiary (fourth) marginal tooth visible above molar prominence in dorsal (Figure 1C) views. Right mandible with apical tooth much longer than first marginal; third marginal nearly symmetrical. Fore-tibia moderately inflated; width:length ratio 0.32. Mesenteric tongue long and tubular forming long mixed segment (Figure 1D). P1 bridged to P3 through a broadly conical EV seating resembling a sunflower receptacle (Figure 3 A–C). Enteric valve cuticle consists of six cushions of unequal size (Figure 4). The largest two cushions comprise a soft basal part covered with fringed scales and 5–6 narrow spines, and bear sclerotized extensions that broaden into wing-like paddles; margins finely pectinate (40–70 compound spines per extension) and approx. a dozen more separated spines in interior of extension (Figure 4). Other cushions soft and scaly, without spines. Middle one, situated between the largest cushions, approx. half their size. Two cushions lateral to the major ones small, approx. half smaller than the middle one. Cushion opposite to the middle one approx. the same length as it, narrower. Soft cushions anchored in their seating and seating lumen filled with bacterial slime; extensions penetrate to middle of P3 lumen (Figure 3C).

**Table 1.** Measurements (in mm) of *Anenteotermescherubimi* workers (n = 18 from 4 colonies).

Measurement	mean	SD
Head length with postclypeus	0.482	0.019
Head width	0.493	0.015
Postclypeus length	0.120	0.007
Pronotum width	0.299	0.016
Hind tibia length	0.366	0.009
Fore-tibia length	0.333	0.015
Fore-tibia width	0.106	0.010
Fore-tibia width/length	0.320	0.033
Total length	2.17	0.23

#### Description of imago.

(male) (Figure 5, Table 2). Tiny, 7 mm. Head with vertex and frons sepia brown, postclypeus slightly lighter. Genae and labrum light brown. Fontanelle ovoid, hyaline. Eyes dark sepia brown. Pronotum lighter than head capsule. Meso- and metanotum slightly lighter than pronotum, with hind margins tinged with yellow. Abdominal tergites and sternites light brown, sternites paler in middle. Wing membrane light brown, with darker veins. Legs light brown. Head capsule rounded posteriorly. Eyes of medium size, protruding. Ocelli elliptical, close to eyes. Antennae with 15 articles. Pronotum slightly broader than long, semi-octogonal, anterior corners deflected downward, hind margin nearly straight. Hind margins of meso- and metanotum narrow, sinuated. Wings long, approx. half their length beyond extremity of abdomen.

**Table 2.** Measurements (in mm) of *Anenteotermescherubimi* male imagos (n = 6 from one colony).

Measurement	mean	SD
Head length to tip of labrum	0.685	0.023
Head length to anterior margin of postclypeus	0.513	0.008
Head width, maximum at eyes	0.591	0.013
Head, interocular width	0.447	0.011
Eye maximum diameter	0.183	0.005
Eye to head base distance	0.031	0.005
Ocellus maximum diameter	0.075	0.005
Ocellus to eye distance	0.017	0.003
Pronotum maximum length	0.407	0.007
Pronotum maximum width	0.515	0.018
Fore wing length from suture	5.89	0.08
Fore wing maximum width	1.51	0.06
Hind tibia length	0.523	0.009
Total length with wings	7.09	0.09
Total length without wings	3.92	0.16

#### Diagnosis.

*Anenteotermescherubimi* is the smallest soldierless termite worker in Africa (head width, HW), followed by *Acidnotermespraus* (0.52 mm HW) and *An.nanus* (0.56 mm HW). The EVA of *A.cherubimi* is very diagnostic and unlike that of any other termite species in having two broad and symmetrical pectinate paddles as opposed to two narrow paddles in other *Aneneteotermes* spp. or tri- and hexa-radial, or asymmetrical armature as in other soldierless genera.

#### Comments.

Sands (1998) placed “*Anenteotermes* new species” in this genus based on a well-developed mixed segment which is only shared in African soldierless workers by *Aderitotermes* and *Adaiphrotermes* (Sands 1972). Of these three genera, only *Anenteotermes* contain species with a single pair of sclerotized EV extensions penetrating into P3. The *Anenteotermes* worker key in Sands (1972, p. 194) can accommodate *An.cherubimi* by inserting a new couplet three as follows and renumbering the subsequent couplets:

## Discussion

Sands (1982) described abdominal autothysis in two live soldierless species from Africa, *Alyscotermeskilimandjaricus* Sands and *Ateuchotermesmuricatus* Sands. Upon grasping an ant with their mandibles, abdominal muscle convulsions were observed causing the integument to split along a line at the back of the metanotum and resulting in expulsion of their hind gut coils. Sands (1982) further noted that *Al.kilimandjaricus* workers secrete droplets of a clear fluid, stored in enlarged salivary glands and released from the dehiscence line before gut expulsion. In *At.muricatus*, the entire intestine of workers was expelled in “a few seconds”. Sands (1982) did not mention a fluid exudate preceding the autothysis. In preserved workers of *An.cherubimi*, it was not possible to confirm a fluid reservoir, but the expulsion of guts from the abdomen was obvious (Figure 2A).

## Supplementary Material

XML Treatment for Anenteotermes cherubimi
